# Editorial: Neuropeptide actions in arthropod biology

**DOI:** 10.3389/fendo.2024.1387176

**Published:** 2024-02-28

**Authors:** Qisheng Song, David Stanley

**Affiliations:** ^1^ Division of Plant Science and Technology, University of Missouri, Columbia, MO, United States; ^2^ Biological Control of Insect Research Laboratory, United States Department of Agriculture-Agricultural Research Station (USDA/ARS), Columbia, MO, United States

**Keywords:** neuropeptide, mechanism, arthropod, hormone, signaling

Animals and plants produce peptides that signal events. The many plant peptides govern aspects of growth and development. *Arabidopsis*, for example, expresses over 1,000 peptides (1). Insects also produce many peptides that operate in a wide range of biology. Antimicrobial peptides (AMPs), for example, are small, mostly positively charged peptides. They are effector molecules with wide range of antibacterial, antifungal and antiparasitic activities. We mentioned them here because they also influence other functions (2). They act in brain functions, such as sleep and non-associative learning. They act in gut homeostasis and in regulating microbiota. The distinction between peptides and neuropeptides is that neuropeptides are produced by neurons. The papers in this Research Topic provide a sense of the manifold neuropeptide actions in insects and other invertebrates.

Cordero-Moline et al. explain neural aspects of female mate choice. Mate choice may be the most important decision(s) females make because off-spring fitness, and hence, the mother’s fitness. The authors pursue several key questions, such as where in the brain does the choice occur? And do behaviors seen in vertebrates, such as sexual cannibalism, also occur in invertebrates? Mating follows several stages, including precopulatory mechanisms that may lead to identification of a potential mate. Female sexual status can influence their copulatory receptivity. Signaling a female’s status can include chemical signals, or moving towards potential mates, among other signals. Females can also respond to stimulation during copulation, which can influence its duration and mating success. Aside from its well-known role in yolk proteins, vitellogenin (Vg) occurs in the brains of several insect species that may represent a larger range of insects. Vg is connected to parental care in the burying beetle, *Nicrophorus vespilloides* and possibly other species.

Some female arthropods have a sperm storage structure called a ‘spermatheca’ which stores sperm transferred to females after copulation, sometimes for years. They can also store sperm from multiple males. Their abilities to regulate spermathecal contractions give females potentials to bias fertilization via nervous control of spermathecae and may give them a mechanism to bias paternity of some offspring, possibly based on cues received from males. Females may also dump sperm in a selective way. Hence, even after selecting a mate by active choice, females express a variety of neural and neuroendocrine mechanisms to manipulate their reproductive success and fitness.

Bursicon (from the Greek “bursikos”, for a tanning process) was originally called a tanning hormone because it was first known to launch a cuticle sclerotization, or tanning, process in newly molted insects. We have since learned that the biological significance of bursicon is considerably broader. It is a heterodimeric neuropeptide hormone composed of two sub-components, bursicon (burs), and partner of burs (pburs). The heterodimer acts in cuticle tanning via its receptor, leucine-rich G protein-coupled receptor 2 in *Drosophila*. The receptor is encoded by the gene, *rickets*. Bursicon can also form burs-burs and pburs-pburs homodimers. An et al. (3) reported the homodimers activate immune and stress genes during molting. More recently, Kong et al. (4) noted that bursicon mediates AMP gene expression during larval crowding as a form of prophylactic immunity in oriental armyworms, *Mythimna separata*.

Yu et al. address still anther bursicon role, now expanded to mediating vitellogeneses and reproduction in female whiteflies, *Bemisia tabaci*. The authors reported that *burs*, *pburs* and *rickets* reached highest expression in newly emerged females, which remained slightly higher through the first few days of adulthood. When the expression of all three genes was knocked down with appropriate RNAi constructs, it led to reduced expression of vitellogenin (Vg) and Vg receptor (VgR). The reduced gene expression led to substantial reductions in several bursicon-regulated reproductive parameters, including juvenile hormone titers, ovarian development, egg deposition, size and hatch. The authors concluded that these three genes, *burs*, *pburs* and *rickets* influence female whitefly reproduction.

Miao et al. broaden the topic with their report on sex reversal in a prawn, *Exopalaemon carinicauda*, an economically important cultured prawn. Like most crustaceans, *E. carinicauda* is a sexually dimorphic species, although monosex cultures lead to higher yield and increased economic value. In the past, monosex cultures were created by manual segregation or manipulating male androgenic glands. Now the authors report on using a CRISPR/Cas9 construct to create heritable all-female cultures. It was known that a differentiation switch gene encodes insulin-like androgenic gland hormone (IAG), which is necessary to produce males. The authors identified and cloned an *E. carinicauda* gene encoding IAG. They used this information to design and create two single-stranded guide RNAs (sgRNAs) which they microinjected into hundreds of embryos at the zygote or first cleavage stages. The authors calculated an editing efficiency of 22.48% for eggs injected at the zygote stage and 13.95% for eggs injected at the first cleavage. Miao et al. drew two conclusions. One, this work provides a new method for monosex population breeding and, two, it opens a way for detailed studies of the natural mechanisms of sexual differentiation in crustaceans.

*Amblyomma* ticks transmit several microbial-linked diseases in Africa and the New World, including rocky mountain spotted fever, babesiosis (infects red blood cells) and, in ruminants, the lethal disease heartwater. These are very dangerous disease vectors and the subjects of serious applied and basic research. Here, Lyu et al. contribute new basic information on neuropeptides that guide specific behaviors needed to shed and get out of exoskeletons. Collectively, these are called ecdysis-related neuropeptides (ERNs). The authors created a catalog of ERNs from 7 spider species and 6 tick species. Their 2D cluster maps show considerable relatedness among spider and tick ERNs. The 3D models of ERNs show color-marked domains of the proteins and string analysis shows connectedness among the proteins. dsRNA injections led to large declines in Burs a and Burs b expression and, functionally, to declines in expression of a selected antimicrobial peptide, defensin. This work contributes new, important basic knowledge on ERNs that has palpable potential for development of a novel, molecular-based tick management technology. Forty years ago, Djerassi et al. (5) recognized a need for new insect management technologies and the very long times required to convert new technologies into practical pest management programs. This work may lead to progress in creating new, more efficacious tick management programs.

Insect immune reactions to infection are launched by several signals, including eicosanoids (6). Eicosanoids are oxygenated derivatives of arachidonic acid (AA), a long-chain 20-carbon fatty acid that occurs in small proportions of the lipids of most insects. Upon bacterial infection, AA and another fatty acid, linoleic acid, can be hydrolyzed from cell membrane phospholipids by several phospholipases A_2_ (PLA_2_). Hrithik et al. reported that bacterial infection stimulates AA biosynthesis in a lepidopteran, *Spodoptera exigua* (7). They identified the genes encoding the elongases and desaturases responsible to converting linoleic acid into AA and showed that cellular immune reactions to bacterial infections depends on the AA biosynthesis. Hrithik et al. report the discovery of four separate PLA_2_s in the Asian onion moth, *Acrolepiopsis sapporensis*. They used transcriptome analysis to identify four PLA_2_ genes, denoted As-PLA_2_A, As-PLA_2_B, As-PLA_2_C and As-PLA_2_D. Phylogenetic analysis indicated these genes encode secretory, or sPLA_2_s, and each PLA_2_ has a separate signal peptide, which also indicates they are sPLA_2_s. The enzymes are expressed in all post-emergence stages, specifically in fat body, hemocytes and gut. The authors show that all four enzymes are inducible by bacterial challenge and all four enzymes act in a specific cellular immune response, formation of melanized nodules. The significance of this work lies in increasing knowledge of PLA_2_s actions in signaling insect cellular immune responses to infections and contributing to the growing recognition of PLA_2_s and eicosanoid signaling in insect immunity.

Dou and Jurenka note that neuropeptides are assorted into families based on amino acid sequences and their functions. Here, they review a select family of neuropeptides – the pyrokinin (PK)/pheromone biosynthesis activating neuropeptide (PBAN) family and their receptors. The gene encoding PBAN produces 5 neuropeptides, among them, PBAN and diapause hormone (DH), responsible for inducing diapause in silkworms, *Bombyx mori*. Neuropeptides in this family act in several functions beyond sex pheromone biosynthesis and diapause, and they occur in most, if not all, insect orders. For a single example, *pban* acts in fire ant, *Solenopsis invicta* biosynthesis of its trail pheromone. Increasing insect transcriptome analyses facilitates discovery of a large number of genes encoding PK/PBAN. Here the authors take care to note that identifying a gene sequence does not, in itself, document biosynthesis of the mature peptide.

Peptides in the PK/PBAN family act in several biological functions beyond biosynthesis of sex pheromones. Examples include melanization in moth larvae, embryonic diapause, puparium formation in the fly, *Neobellieria bullata*, and ending pupal diapause in heliothine moths. Within the Lepidoptera, PK/PBAN peptides also act in ending larval diapause in the bamboo worm, *Omphisa fuscidentalis*. The authors list other functions, as well. While these are pleiotropic peptides, it is interesting to consider how such specialized function evolved in a small number of species.

The authors considered the location of PBAN within the central nervous system. They review the work of various scientists who used immunohistochemistry to find PBAN in some cells of the corpora cardiaca, thoracic ganglia and abdominal ganglia in moths. PBAN locations seem similar also in *Drosophila*, locusts, cockroaches and fire ant CNS, but not in the terminal abdominal ganglion. They also considered receptors for the PK/PBAN, all G-protein coupled receptors. The receptors are assorted into two groups, based on the peptide sequences they bind. PBAN receptors occur in most insect orders and can be activated by very low PBAN concentrations.

Dou and Jurenka widely broaden our understanding of PK/PBAN neuropeptides and invite continue research into these biologically important factors.

## Author contributions

QS: Writing – original draft, Writing – review & editing. DS: Writing – original draft, Writing – review & editing.
